# Dose-response effect of *Bifidobacterium lactis* HN019 on whole gut transit time and functional gastrointestinal symptoms in adults

**DOI:** 10.3109/00365521.2011.584895

**Published:** 2011-06-13

**Authors:** Philip A Waller, Pramod K Gopal, Gregory J Leyer, Arthur C Ouwehand, Cheryl Reifer, Morgan E Stewart, Larry E Miller

**Affiliations:** 1 Accurate Clinical Research, Houston, TX, USA; 2 Digestive and Immune Health, Fonterra Research Centre, Palmerston North, New Zealand; 3 Danisco USA, Health and Nutrition, Madison, WI, USA; 4 Danisco Finland, Health and Nutrition, Kantvik, Finland; 5 Sprim Advanced Life Sciences, San Francisco, CA, Sprim, USA

**Keywords:** Bifidobacterium lactis HN019, gastrointestinal, probiotic, whole gut transit time

## Abstract

***Objective.*** To assess the impact of *Bifidobacterium lactis* HN019 supplementation on whole gut transit time (WGTT) and frequency of functional gastrointestinal (GI) symptoms in adults. ***Material and methods.*** We randomized 100 subjects (mean age: 44 years; 64% female) with functional GI symptoms to consume a proprietary probiotic strain, *B. lactis* HN019 (Fonterra Research Centre, Palmerston North, New Zealand), at daily doses of 17.2 billion colony forming units (CFU) (high dose; *n* = 33), 1.8 billion CFU (low dose; *n* = 33), or placebo (*n* = 34) for 14 days. The primary endpoint of WGTT was assessed by X-ray on days 0 and 14 and was preceded by consumption of radiopaque markers once a day for 6 days. The secondary endpoint of functional GI symptom frequency was recorded with a subject-reported numeric (1–100) scale before and after supplementation. ***Results.*** Decreases in mean WGTT over the 14-day study period were statistically significant in the high dose group (49 ± 30 to 21 ± 32 h, *p* < 0.001) and the low dose group (60 ± 33 to 41 ± 39 h, *p* = 0.01), but not in the placebo group (43 ± 31 to 44 ± 33 h). Time to excretion of all ingested markers was significantly shorter in the treatment groups versus placebo. Of the nine functional GI symptoms investigated, eight significantly decreased in frequency in the high dose group and seven decreased with low dose, while two decreased in the placebo group. No adverse events were reported in any group. ***Conclusions.*** Daily *B. lactis* HN019 supplementation is well tolerated, decreases WGTT in a dose-dependent manner, and reduces the frequency of functional GI symptoms in adults.

## Introduction

Functional gastrointestinal symptoms are nonspecific conditions with no identifiable structural or biochemical cause [1] that often present concomitantly [2]. The burden of functional gastrointestinal symptoms is enormous as 42–69% of the population report at least one of these disorders [3,4]. Treatment is complicated by the non-specific presentation of symptoms and by the fact that 75% of people do not seek medical care when symptoms arise [5]. Therefore, management of functional gastrointestinal symptoms is generally self-prescribed and directed at treating individual symptoms. Despite acceptable short-term symptom amelioration with over-the-counter and homeopathic remedies in many cases, no known treatment for functional gastrointestinal symptoms is known to be safe and effective over the long term.

Functional gastrointestinal symptoms are often associated with disturbed intestinal transit. Whole gut transit time (WGTT) significantly influences enterohepatic bile acid and steroid hormone circulation as well as colonic pH and short-chain fatty acid concentrations. Thus, it has been postulated that functional gastrointestinal symptoms may be associated with an increased risk of gallstones, and possibly bowel and breast cancer, when presenting concomitantly with prolonged WGTT [6].

There is a clear need for treatments for functional gastrointestinal symptoms and intestinal transit disturbances that are safe, effective, and sustainable. There is a growing consumer interest in functional foods that can either promote the state of well being and/or alleviate symptoms of certain medical conditions. Probiotics may offer a promising alternative that can be offered in a food or dietary supplement format that is appealing to consumer preferences. Probiotics have been extensively studied in the field of gastroenterology and show potential for improving gastrointestinal symptoms and reducing WGTT [7-11]. However, the benefit of probiotic supplementation is believed to be strain specific and, therefore, clinical trials are required to confirm the safety and efficacy of individual strains.

*Bifidobacterium lactis* HN019 is a probiotic strain for which many health benefits have been established, primarily related to immune enhancement [12]. *B. lactis* HN019 has also been shown to confer beneficial changes to the intestinal microflora [13] although no studies have examined the effects of *B. lactis* HN019 on functional gastrointestinal symptoms or WGTT. This study was designed to determine in a triple-blind, randomized, placebo-controlled trial if dietary supplementation of *B. lactis* HN019 can shorten WGTT and reduce the frequency of functional gastrointestinal symptoms in adults.

## Material and methods

This single-center, triple-blind, randomized, placebo-controlled, dose-ranging study was conducted at Accurate Clinical Research (Houston, TX, USA). All research procedures performed in this trial were in strict accordance with a pre-defined protocol that was approved by all researchers. IntegReview Ethical Review Board (Austin, TX, USA) approved the study protocol on 11 August 2009 and participants provided informed consent before participation. This study is registered under ClinicalTrials.gov number NCT01171014.

### Trial design

This was a single center, age- (25–50 years and 51–65 years) and gender-stratified, triple-blind, placebo-controlled, parallel-group, dose-ranging study with a 1:1:1 allocation ratio among three study groups.

### Participants

Eligible subjects included males and females aged 25–65 years; self report of stool type 2–4 on the Bristol Stool Chart; and average of 1–3 bowel movements per week. Exclusion criteria were use of any probiotic product intended to improve gastrointestinal function within the 2 weeks preceding study entry; major chronic and uncontrolled systemic medical conditions; severe gastrointestinal conditions known to prolong WGTT; lactose intolerance; chronic diarrhea; gastric bypass surgery or lap band insertion for weight loss; regular laxative use; and pregnant or breast-feeding women.

### Interventions

Study products (*B. lactis* HN019 and placebo powders) were supplied in capsules by Danisco USA, Inc. (Madison, Wisconsin, USA). The capsules contained either *B. lactis* HN019 at one of two dose levels (17.2 billion colony forming units (CFU) or 1.8 billion CFU) or placebo. The excipient used to adjust the bacteria count per capsule and to serve as the placebo was rice maltodextrin. All products were stored refrigerated at the study site until time of use and were refrigerated by the participants throughout the supplementation period. Product stability was monitored during the study and no significant reduction in viable counts was observed.

**Figure 1:**
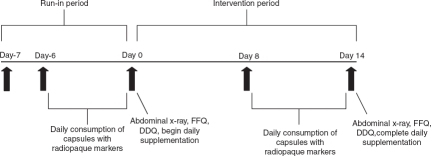
Timeline of study activities. DDQ = digestive discomfort questionnaire; FFQ = food frequency questionnaire.

A timeline of study activities is presented in Figure 1. Following screening procedures, subjects began a 1-week run-in period during which the normal diet was consumed without taking any study product. During this period, subjects were asked to discontinue the consumption of laxatives, dietary fiber supplements, probiotics including *B. lactis,* and any other products that might affect WGTT. Following the run-in period, subjects were randomized into one of three study groups: 17.2 billion CFU *B. lactis* HN019 per day (high dose, *n* = 33), 1.8 billion CFU *B. lactis* HN019 per day (low dose, *n =* 33), or placebo (*n* = 34). Over 14 consecutive days, study products were consumed once daily with breakfast by adding the entire contents of a capsule to commercial yogurt that was free from probiotics (product contained standard yogurt starter cultures *Lactobacillus bulgaricus* and *Streptococcus thermophilus).*

The classic single film estimate was utilized to quantify WGTT [14]. Each subject ingested 24 radiopaque markers per day for six consecutive days prior to abdominal X-rays on days 0 and 14. The number of markers present in the right, left, and rectosigmoid colon were summed to yield a total marker count. Marker counts were identified by a single board-certified radiologist (Gulf Coast MRI & Diagnostics, Houston, TX, USA) who was blinded to subject treatment assignment.

### Outcomes

The primary endpoint of WGTT was assessed by abdominal X-ray on days 0 and 14. A food frequency questionnaire was completed on days 0 and 14 to determine diet consistency during the study period. Data were collected on the frequency of ingestion of 17 food categories over the previous week including dairy products (milk, cheese, yogurt, and ice cream); meat, fish, or poultry; eggs; peanut butter or nuts; citrus fruits or juices; dark green leafy or deep orange vegetables; other fruits or vegetables; bread, cereal, rice, or pasta; sweets; salty snacks; soft drinks; alcohol; coffee or tea; and fast food. The frequency of food consumption in each category was self-rated on a 5-point Likert scale (0 = never, 1 = seldom, 2 = 2–3 times per week, 3 = once a day, and 4 = more than once a day). A questionnaire was administered on study days 0 and 14 to evaluate the frequency of nine upper (vomiting, regurgitation, abdominal pain, nausea, and gurgling) and lower (constipation, diarrhea, irregular bowel movements, and flatulence) gastrointestinal symptoms experienced over the previous week. The frequency of each symptom was subject-reported using a numeric scale anchored at 1 (never) and 100 (always). Adverse events were defined as any untoward medical occurrence that occurred during the trial, regardless of the relationship with the administered study product.

### Sample size

A total required sample size of 100 subjects equally distributed among the three treatment groups was calculated based on an anticipated 33% reduction in WGTT versus placebo for either probiotic group, a 20% difference between the two probiotic groups, a 9% attrition rate, a two-sided alpha level of 0.05, and 87% statistical power.

### Randomization

Randomization was stratified by age (25–50 years or 51–65 years) and gender and was implemented using a permuted block design. Subjects were randomly allocated to one of the three treatment groups within their age- and gender-stratification categories using four computer-generated randomization lists. The blinded randomization sequence was generated by a biostatistician at Sprim Advanced Life Sciences, Sprim USA (San Francisco, CA, USA). The study coordinator at the investigative site enrolled and assigned subjects to treatment groups. Study products were labeled with sequential subject identification numbers within each stratum and were provided to the site by the sponsor. The site was instructed to enroll subjects consecutively within the appropriate age/gender stratum.

### Blinding

The study was conducted using triple-blinding methods: subjects, investigators, outcome assessors, study site personnel, data managers, biostatisticians, and the sponsor remained blinded to the treatment assignments until data analyses were completed. Study products were delivered to the investigative site in identical containers labeled only with the lot number and subject identification codes. All capsules were identical in appearance, texture, taste, and smell.

### Statistical methods

Data were recorded on case report forms and double-entered into a database that was verified and independently monitored for accuracy by Sprim Advanced Life Sciences, Sprim USA. Baseline subject characteristics for the three treatment groups were reported using mean and standard deviation for intervally and ordinally scaled variables and frequencies and percentages for categorical variables. Comparisons of change from study days 0–14 across treatment groups were performed using oneway analysis of variance (ANOVA). When a resulting ANOVA *p*-value was <0.05, *post hoc* tests were conducted to determine which groups were statistically different from each other at an alpha level of 0.05.

WGTT was calculated using the formula:


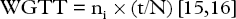


where n_i_ is the number of markers observed on X-ray, t is the time between marker ingestions in hours, and N is the total number of markers ingested each day. Thus, in this study, t/N equals 1 (24 markers per capsule/24 h between marker ingestions), and WGTT is therefore equal to the total marker count. Changes in WGTT between study days 0 and 14 were compared across treatment groups using ANOVA. Analysis of covariance (ANCOVA) was used to remove the effect of potentially confounding baseline differences among the groups and determine the significance of the effect of treatment on WGTT independent of the potential confounders gender, age, race, and baseline WGTT. The Kaplan-Meier method was used to evaluate time to excretion of all markers by treatment on days 0 and 14 and the significance of inter-group differences was assessed using the Mantel-Cox test. The Wilcoxon signed-rank test was used to evaluate changes in gastrointestinal symptom frequency for each group. Statistical analyses were performed using BMDP software (Version 8.2, Statistical Solutions, Saugus, MA, USA).

## Results

### Participant flow

**Figure 2:**
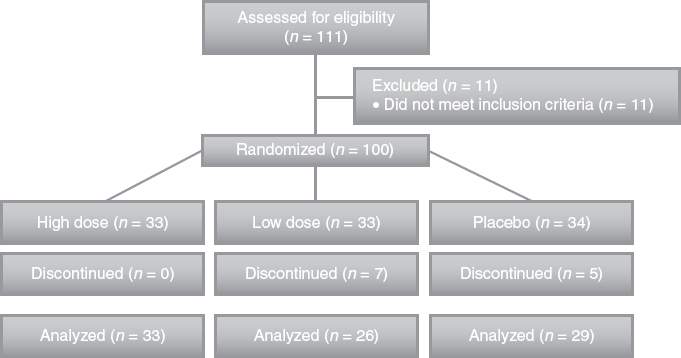
CONSORT patient flow diagram. High dose, 17.2 billion CFU *Bifidobacterium lactis* HN019; Low dose, 1.8 billion CFU *B. lactis* HN019. CFU = colony forming unit.

A total of 100 subjects were enrolled and randomized. Abdominal X-ray endpoint data were available at days 0 and 14 for 88 subjects (Figure 2). Paired gastrointestinal symptom and food frequency data were available for 87 subjects. All subjects with data for both study days 0 and 14 were used in the analysis. No subject withdrawals were related to the study product. Among subjects who completed the study, compliance with the study product was 100% in each study group.

### Recruitment

Subjects were enrolled in this clinical study between June 2009 and November 2009 and subject follow-up continued through December 2009.

### Baseline data

Baseline subject characteristics were similar among the three treatment groups (Table I). Frequency of consumption of all food categories was, in general, similar across the treatment groups. Functional gastrointestinal symptom frequency was comparable across groups with constipation, irregular bowel movements, and flatulence most commonly reported. Total WGTT was comparable among the three treatment groups. Across all groups, females (55 ± 34 h) had a significantly longer (*p* = 0.02) baseline WGTT versus males (41 ±24 h) while race and body mass index had no influence on WGTT.

**Table I:** Baseline subject characteristics.

Characteristic	High dose (*n* = 33)	Low dose (*n* = 26)	Placebo (*n* = 29)	*P*
Age, yr (mean ± sd)	43 ± 12	44 ± 11	45 ± 11	0.78
Female gender, *n* (%)	20 (61)	16 (62)	20 (69)	0.76
Ethnicity, *n* (%)				0.64
-African American	19 (58)	16 (61)	18 (62)	
-Hispanic	7 (21)	7 (27)	4(14)	
-White	6(18)	3 (12)	7(24)	
-Asian	1 (3)	0	0	
Body mass index, kg/m^2^ (mean ± sd)	32 ± 8	30 ± 7	32 ± 8	0.73
Whole gut transit time, h (mean ± sd)				
-Right colon	17 ± 11	22 ± 15	13 ± 9	0.04
-Left colon	17 ± 15	23 ± 17	17 ± 19	0.18
-Rectosigmoid	15 ± 14	16 ± 11	11 ± 12	0.19
-Total	49 ±30	60 ±33	42 ± 31	0.13
GI symptom frequency (mean ± sd)*				
-Upper GI symptoms				
-Vomiting	14.8 ± 21.7	11.0 ± 20.2	8.0 ± 18.6	0.44
-Regurgitation	30.7 ± 27.7	24.9 ± 28.7	18.4 ± 22.6	0.20
-Abdominal pain	34.6 ± 26.6	43.8 ± 30.6	36.0 ± 29.6	0.44
-Nausea	28.6 ± 24.7	29.9 ± 26.8	23.2 ± 26.8	0.60
-Gurgling	47.7 ± 30.9	45.1 ± 29.4	38.3 ± 26.4	0.45
-Lower GI symptoms				
-Constipation	68.7 ± 22.0	75.7 ± 30.4	60.9 ± 32.5	0.16
-Diarrhea	14.8 ± 20.0	9.8 ± 21.9	23.2 ± 31.0	0.13
-Irregular bowel movements	61.7 ± 28.8	61.7 ± 32.0	53.8 ± 36.3	0.57
-Flatulence	59.7 ± 32.8	49.0 ± 32.6	46.7 ± 34.0	0.26

Abbreviations: GI = gastrointestinal; sd = standard deviation;

* self-rated on a numeric scale anchored at 1 = never and 100 = always.

### Numbers analyzed

A total of 100 subjects were enrolled and randomized. Of these subjects, 0 in the high dose group, 7 in the low dose group, and 5 in the placebo group discontinued before the day 14 study visit. All 88 subjects with WGTT data and 87 subjects with gastrointestinal symptom frequency data for study days 0 and 14 were included in the analysis.

### Outcomes and estimation

*Food frequency.* When changes in food frequency over the study period were evaluated, each specific food type was consumed with comparable frequency across study groups throughout the study with the exception of yogurt consumption (data not shown). Since the study product was consumed in yogurt, the median yogurt intake significantly increased in all three study groups from never (low dose) or seldom (high dose and placebo) at day 0 to once a day (all groups) at day 14. Overall, food consumption habits among groups were similar and likely had no influence on the primary study outcomes.

### Whole gut transit time

**Figure 3:**
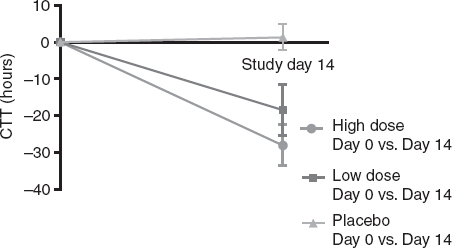
Change in whole gut transit time over 14 days by study group. Values represent mean ± 95% confidence interval. High dose, 17.2 billion CFU *Bifidobaaerium lactis* HN019; Low dose, 1.8 billion CFU *B. lactis* HNO19. CFU = colony forming unit.

Both *B. lactis* HN019 groups had a statistically significant decrease in mean WGTT over the 14-day study period (high dose = 33% reduction, low dose = 25% reduction) with no change observed in the placebo group (Figure 3). The difference in WGTT change was statistically significant (*p* < 0.001) across study groups, thereby representing a strong dose-response effect of *B. lactis* HN019 (Table II).

**Table II:** Whole gut transit time by treatment group and study day.

Whole gut transit time	High dose (*n* = 33)	Low dose (*n* = 26)	Placebo (*n* = 29)
Day 0, mean h	49.2	59.5	42.5
95% CI	39.2 to 59.2	46.7 to 72.3	31.3 to 53.7
Min, median, max	0, 49, 135	9, 68, 111	0, 35, 131
Day 14, mean h	21.0	41.0	43.8
95% CI†	-	-	-
Min, median, max	0, 5, 131	0, 26.5, 141	0, 46, 131
Absolute change, mean h*	-28.1	-18.5	1.3
95% CI	-38.9 to -17.3	-32.3 to -4.6	-5.7 to 8.3
Min, median, max	-87, -33, 65	-84, -18, 67	-35, 5, 39
Relative change, mean %*	-32.6‡	-24.5	16.7‡
95% CI†	-	-	-
Min, median, max	-100, -94, 600	-100, -48, 214	-100, 11, 286

Abbreviation: CI = confidence interval;

* *p* < 0.001 among groups;

† CIs were not calculated when distributions were non-normal;

‡ relative change could not be calculated for a denominator of 0, resulting in exclusion of one subject each from the high dose and placebo groups.

Due to differences in baseline WGTT among the three treatment groups, an ANCOVA model was used to examine inter-group differences in WGTT at day 14 adjusted for baseline WGTT differences in order to minimize possible confounding effects. After controlling for baseline levels, WGTT at day 14 remained significantly different (*p* = 0.001) among the three study groups.

Figure 4a shows Kaplan-Meier curves for time to excretion of all ingested markers at baseline, which were not significantly different among the three treatment groups. Following 14 days of supplementation with *B. lactis* HN019, the high dose group had a significantly shorter WGTT than both the low dose and placebo groups (*p* < 0.05) (Figure 4b).

**Figure 4a:**
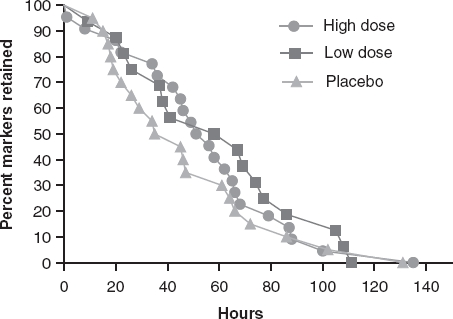
Time to radiopaque marker excretion at pre-treatment by study group. High dose, 17.2 billion CFU *Bifidobacterium lactis* HNO 19; Low dose, 1.8 billion CFU *B. lactis* HN019. CFU = colony forming unit.

**Figure 4b:**
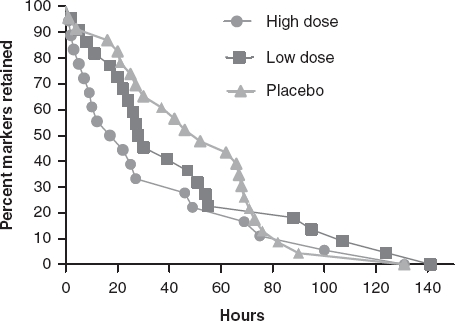
Time to radiopaque marker excretion at 14 days post-treatment by study group. High dose, 17.2 billion CFU *B. lactis* HNO 19; Low dose, 1.8 billion CFU *B. lactis* HNO 19. CFU = colony forming unit.

### Functional gastrointestinal symptom frequency

Statistically significant improvements in most gastrointestinal symptom frequency scores were reported between days 0 and 14 in the high and low dose groups. Most symptoms improved in the high dose (eight of nine) and low dose (seven of nine) groups, respectively, while only two of nine symptoms showed a statistically significant improvement with placebo (Table III). Of particular interest were the changes in constipation, irregular bowel movements, and flatulence since symptoms were reported with the highest frequency at baseline. For each of these symptoms, the relative decrease in symptom frequency was approximately two-fold greater in the *B. lactis* HNO 19 groups compared to placebo.

**Table III:** Mean absolute change in GI symptom frequency score over the 14-day supplementation period.

Symptom*	High dose (*n* = 33)	Low dose (*n* = 26)	Placebo (*n* = 28)
Upper GI symptoms			
Vomiting	-9.8*	-7.4	-2.2
Regurgitation	-14.9*	-11.3*	-2.3
Abdominal pain	-17.9†	-26.6‡	-8.2
Nausea	-13.8*	-13.8†	-3.5
Gurgling	-12.9*	-23.9‡	-4.7
Lower GI symptoms			
Constipation	-29.0‡	-35.8‡	-14.2*
Diarrhea	-2.9	0.0	-9.4*
Irregular bowel movements	-19.0†	-22.8†	-9.5
Flatulence	-14.0*	-15.3*	-5.9

Abbreviation: GI = gastrointestinal;

* self-rated on a numeric scale anchored at 1 = never and 100 = always; ^*^p < 0.05

† *p* < 0.01,

‡ *p* < 0.001,

### Harms

Aside from functional gastrointestinal symptoms reported on the questionnaire, no adverse events were reported in any group during the study.

## Discussion

This randomized, triple-blind, placebo controlled, dose-ranging study provides Level I evidence that dietary consumption of *B. lactis* HNO 19 shortens WGTT in a dose-dependent manner and reduces the frequency of functional gastrointestinal symptoms in adults. In addition, the absence of reported adverse events suggests that short-term *B. lactis* HN019 supplementation is safe. The role of probiotics in influencing intestinal disturbances, in particular, preventing or shortening the duration of diarrhea in infants or adults with acute gastroenteritis is well established [17-20]. The effect of probiotics on intestinal transit has been studied to a limited extent although, aside from the current trial, no study has shown an improvement in WGTT.

Several potential mechanisms have been suggested that may explain the effect of a probiotic strain on intestinal motility [21,22]. These mechanisms include an increase in fecal bacterial mass, stimulation of cholecytokinin, and deconjugation of bile salts resulting in free secondary bile salts that can stimulate colonic motility and excretion. In a small clinical trial with 32 volunteers, Marteau et al. [22] showed consumption of *B. lactis* DN-173 010 shortened WGTT in women aged 18–45 years. This effect was not due to deconjugation or dehydroxylation of bile salts. The authors speculated that a product of bacterial origin may decrease sigmoid tonus and stimulate colonic motility. Phloroglucinol is one such compound that is known to have strong effect on colonic motility [23,24]. However, we did not analyze fecal metabolites in the current study to validate this hypothesis. Supplementation with *B. lactis* HN019 has been shown to increase the level of resident bifidobacteria and lactobacilli and to reduce enterobacteria counts in adults [13]. This may provide a further potential mechanism of action for *B. lactis* HN019 supplementation since increased levels of lactic acid-producing bacteria may lower the colonic pH and production of other short-chain fatty acids may stimulate peristalsis and potentially result in a shorter WGTT [25].

The clinical importance of a short-term decrease in WGTT is subject to debate. However, it is reasonable to assume that if long-term probiotic consumption could chronically lower WGTT, this may potentially yield a meaningful decrease in the risk of associated colorectal conditions, such as colon cancer. The beneficial effect of daily *B. lactis* HN019 on WGTT is at least equivalent to that of dietary fiber. However, unlike fiber, *B. lactis* HN019 also improves gastrointestinal symptoms [26-28]. Furthermore, *B. lactis* HN019 is safe for human consumption as no untoward side effects associated with the product have been reported, a finding that is well established including data from studies with infants to elderly [12,29].

Subjects in the present study suffered from functional gastrointestinal symptoms with constipation, irregular bowel movements, and flatulence as the predominant symptoms. The outcomes of this study suggest that *B. lactis* HN019 supplementation reduces the frequency of many common upper and lower gastrointestinal symptoms. Since these symptoms are generally non-specific, management strategies are narrowly focused on the treatment of individual symptoms. *B. lactis* HN019 is potentially advantageous since this single product may be used to alleviate multiple gastrointestinal symptoms simultaneously. Although the present study utilized capsules, fermented and non-fermented dairy products are an ideal matrix for the inclusion and consumption of *B. lactis* HN019.

The present study has several strengths including randomized treatment allocation, use of placebo controls, assessment of dose ranging efficacy, evaluation of dietary intake during the study, and use of stringent data collection and blinding methods. This study also had several limitations. The results presented herein are applicable only to *B. lactis* HN019 and cannot be generalized to other probiotic strains or products. Caution should be exercised in extrapolating these study outcomes to people with chronic and/or severe gastrointestinal complications. A final limitation of the study was that almost one in three subjects in the probiotic groups had no observable markers on follow-up radiographs, which indicated a WGTT of less than 24 h. However, according to the formula used to calculate WGTT [15,16], these subjects were assigned a WGTT of 0 h, a value that is physiologically impossible. Nevertheless, the conclusions of this trial are valid since nonparametric statistical methods corroborated the findings of the primary analysis.

In conclusion, consumption of the probiotic *B. lactis* HN019 is well tolerated, decreases WGTT in a dose-dependent manner, and reduces the frequency of functional gastrointestinal symptoms in adults.
